# Cost-Utility of Acromegaly Pharmacological Treatments in a French Context

**DOI:** 10.3389/fendo.2021.745843

**Published:** 2021-10-07

**Authors:** Thierry Brue, Philippe Chanson, Patrice Rodien, Brigitte Delemer, Delphine Drui, Lucile Marié, Laurène Juban, Lara Salvi, Robin Henocque, Gérald Raverot

**Affiliations:** ^1^ Assistance Publique-Hôpitaux de Marseille (AP-HM), Department of Endocrinology, Hôpital de la Conception, Centre de Référence des Maladies Rares de l’hypophyse HYPO, Marseille, France; ^2^ Aix-Marseille Université, Institut National de la Santé et de la Recherche Médicale (INSERM), U1251, Marseille Medical Genetics (MMG), Institut Marseille Maladies Rares (MarMaRa), Marseille, France; ^3^ Université Paris-Saclay, Inserm, Physiologie et Physiopathologie Endocriniennes, Assistance Publique-Hôpitaux de Paris, Hôpital Bicêtre, Service d’Endocrinologie et des Maladies de la Reproduction, Centre de Référence des Maladies Rares de l’Hypophyse, Le Kremlin-Bicêtre, France; ^4^ Université d’Angers, CHU d’Angers, service d’Endocrinologie-Diabétologie-Nutrition, Centre de Référence des Maladies Rares de l’Hypophyse, Angers, France; ^5^ CHU de Reims - Hôpital Robert Debré, Service d’Endocrinologie – Diabète – Nutrition, Reims Cedex, France; ^6^ Endocrinology Department, L’institut du thorax, University Hospital of Nantes, Nantes Cedex, France; ^7^ Stève Consultants, Paris, France; ^8^ Rare Disease, Pfizer France, Paris Cedex, France; ^9^ Endocrinology Department, “Groupement Hospitalier Est” Hospices Civils de Lyon, Bron, France

**Keywords:** acromegaly, cost-utility, growth hormone, insulin-like growth factor-1, QALY, pasireotide, pegvisomant, somatostatin

## Abstract

**Objective:**

Efficacy of pharmacological treatments for acromegaly has been assessed in many clinical or real-world studies but no study was interested in economics evaluation of these treatments in France. Therefore, the objective of this study was to estimate the cost-utility of second-line pharmacological treatments in acromegaly patients.

**Methods:**

A Markov model was developed to follow a cohort of 1,000 patients for a lifetime horizon. First-generation somatostatin analogues (FGSA), pegvisomant, pasireotide and pegvisomant combined with FGSA (off label) were compared. Efficacy was defined as the normalization of insulin-like growth factor-1 (IGF-1) concentration and was obtained from pivotal trials and adjusted by a network meta-analysis. Costs data were obtained from French databases and literature. Utilities from the literature were used to estimate quality-adjusted life year (QALY).

**Results:**

The incremental cost-utility ratios (ICUR) of treatments compared to FGSA were estimated to be 562,463 € per QALY gained for pasireotide, 171,332 € per QALY gained for pegvisomant, and 186,242 € per QALY gained for pegvisomant + FGSA. Pasireotide seems to be the least cost-efficient treatment. Sensitivity analyses showed the robustness of the results.

**Conclusion:**

FGSA, pegvisomant and pegvisomant + FGSA were on the cost-effective frontier, therefore, depending on the willingness-to-pay for an additional QALY, they are the most cost-effective treatments. This medico-economic analysis highlighted the consistency of the efficiency results with the efficacy results assessed in the pivotal trials. However, most recent treatment guidelines recommend an individualized treatment strategy based on the patient and disease profile.

## Introduction

Acromegaly is a rare disease characterized by progressive somatic disfigurement, mainly involving the face and extremities, together with systemic manifestations related to organ overgrowth (1). The most common cause of the disease is the presence of a benign tumor or adenoma originating from pituitary somatotroph cells and secreting excess growth hormone (GH) (2, 3). This excessive secretion of GH leads to a persistent elevation of IGF-1, which facilitates the growth-promoting effects of GH (4). Manifestations of acromegaly include tissue overgrowth, joint pain and deformation, hypertension, metabolic impairment and heart and respiratory failure (5–8).

Recent studies suggest that the prevalence of acromegaly would be around 94 cases per 100,000 inhabitants from a study performed in Belgium and 1,034 per million from a systematic biochemical study performed in Germany (9, 10). In 2017, the Acromegaly Consensus Group updated the most recent consensus guidelines on the medical management of acromegaly. Surgical resection of the pituitary adenoma is considered as the gold-standard therapy and represents the optimal opportunity for cure. For patients for whom surgery is not possible or with a persistent disease, first-generation somatostatin analogs (FGSA) are recommended as first-line treatment. In case of inadequate GH and/or IGF-1 control with FGSA, the treatment should be individualized. Pasireotide is recommended in second-line treatment if the residual tumor is still present and the resection is unsuitable. Patients with impaired glucose history or hyperglycemia occurrence switch to pegvisomant. Adding pegvisomant to FGSA is recommended in case of clinically relevant residual tumor and pre-existing impaired glucose metabolism (11).

Few studies assessed the cost-effectiveness or cost-utility of acromegaly treatments but none were based on French data (12–18). The objective of this analysis was to assess the cost-utility of second-line treatments in patients with inadequate response to surgery and/or radiation therapy and in whom an appropriate medical treatment with FGSA did not normalize IGF-I concentrations or was not tolerated.

## Materials and Methods

### Model Structure

**Figure 1:**
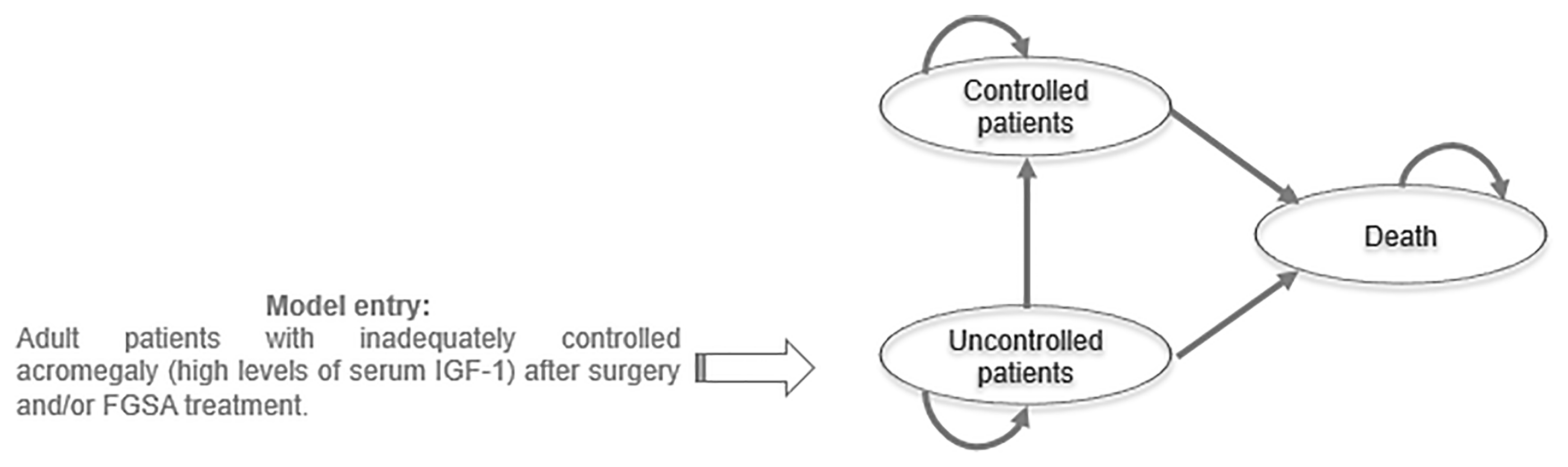
Model structure.

A Markov model was developed in Microsoft Excel (2016) to simulate the lifetime disease progression of a cohort of 1,000 patients inadequately controlled by FGSA and/or surgery. This model included three health states based on IGF-1 normalization and death (**Figure 1**). IGF-1 normalization was selected as criterion for the model since this is the only common criterion between all treatments of interest. In most clinical studies in acromegaly, treatment effects were assessed at least 12 weeks after treatment initiation. However, some patients needed more time to be controlled (e.g. treatment effects could be delayed). Therefore, 12-week cycles were applied during the first year. Treatment effects were assessed every 12 weeks until the end of the 1st year. Therefore, during the 1st year, patients who normalized IGF-1, based on treatment efficacy data, moved to the “Controlled patients” state and continued treatment over the time horizon. For patients who stayed into the “Uncontrolled patients” state after one year, the same treatment was maintained over the time horizon and no treatment effect was applied.

To cover the lifetime progression of the disease, patients entered into the model at the median age at diagnosis (47 years old) and were followed during 40 years. Future costs and health effects were discounted to reflect their present value by using French social discount rate of 2.5%.

Treatments included in the analysis were based on most recent clinical treatment guidelines (11). Therefore, for the second-line analysis, pasireotide, pegvisomant and pegvisomant with FGSA (off label) were compared. In addition, FGSA alone was included as a comparator even if FGSA are not tolerated for patients of interest, but in real-life context, they could be used by these patients.

### Transition Probabilities

Efficacy of treatments in the model was defined as the normalization of IGF-1 concentration. Since no direct comparison between all strategies was available, a network meta-analysis (NMA) has been performed with pasireotide as the reference treatment to generate adjusted comparisons with all treatments of interest (**Table 1**). The NMA was based on the pivotal clinical trials (19–23). For Trainer et al., 2000, only the results for the 15 mg arm were included in the NMA to fit with the mean dose of pegvisomant during the first year of treatment in real-life (19, 24).

**Table 1:** Treatment efficacy data and NMA results.

	Parameter Value	Reference
Treatment efficacy – 2^nd^ line		
Efficacy data included in the NMA		
Pegvisomant (15mg)	75.0%	Trainer et al. (19)
Pegvisomant (10 mg)	56.0%	Trainer et al. (20)
Pegvisomant (10 mg) and FGSA	61.5%	
Pasireotide (50 mg qd) *^╪^*	25.4%	Gadelha et al. (21)
Pasireotide (400 µg bid)	25.9%	Petersenn et al. (22)
FGSA	20.3%	
Efficacy data of pasireotide as the reference treatment in the model		
At 12 weeks	12.7%	Gadelha et al. (21)
At 24 weeks	25.4%	
At 36 weeks	30.9%	Gadelha et al. (23)
At 48 weeks	33.0%	
Hazard ratio used for other treatments		
Pegvisomant	4.6	NMA
Pegvisomant and FGSA	5.3	
FGSA	0.3	

^╪^Results from 40mg and 60mg arms have been combined.

Since pasireotide is the only treatment with efficacy data available at different time points of the first year, it was selected as the reference treatments and hazard ratios from the NMA were applied to estimate the efficacy inputs used in the model (**Table 2**).

**Table 2:** Treatment efficacy used in the model.

Treatments	Proportion of patients with IGF-1 normalized				
	Baseline	At 3 months	At 6 months	At 9 months	At 12 months
Pasireotide	0%	12.69%	25.38%	30.89%	32.95%
FG SA	0%	0.19%	1.57%	2.85%	3.46%
Pegvisomant	0%	54.49%	66.81%	70.79%	72.15%
Pegvisomant + FGSA	0%	65.34%	75.38%	78.49%	79.54%

FGSA, First-generation somatostatin analogs; IGF-1, Insulin-like growth factor-1.

Depending on the health state “Controlled patients” or “Uncontrolled patients”, a standardized mortality ratio (SMR) of respectively 1.1 or 2.5 was applied on age and gender general French population distribution from the Institut national de la statistique et des études économiques (INSEE) (25).

### Treatment Safety

Adverse events (AE) associated to treatment were included in the model because they might have had a significant impact on the cost-utility results, especially due to the cost associated with their management. AE incidence rates are reported in **Table 3**.

**Table 3:** Treatment safety inputs.

Treatment safety – 2^nd^ line	Parameter Value	Reference
Pegvisomant		
Diarrhoea	6.25%	Trainer et al. (19)
Flatulence	5.00%	
Nausea	7.50%	
Pasireotide		
Diarrhea	15.20%	Gadelha et al. (21)
Hyperglycemia	9.60%^╪^	
Diabetes mellitus	20.80%/1.60%^╪^	
Abdominal Pain	0.80%^╪^	
Pegvisomant and FGSA		
NA	No treatment-related reported in any publications	
FGSA		
Diarrhea	1.52%	Gadelha et al. (21)
Diabetes mellitus	4.55%	

^╪^Grade 3/4; FGSA, First-generation somatostatin analogs; NA, Not Applicable.

### Health-Related Quality-of-Life Estimates

Since no consensus about the effect of biochemical control on quality of life in acromegaly has been reached utilities of the general population were used for controlled patients in the model (26–28). For uncontrolled patients, a relative decrease of utility due to acromegaly was estimated from Rowles et al. In this study, median utility of acromegaly patients was estimated at 0.70 while general population was 0.81, therefore the disutility due to acromegaly was estimated at 20.5% (29) (**Table 4**).

**Table 4:** Utility and disutility inputs.

Item	Parameter	Source
Utility values		
Controlled		
45-54 yo	0.922	Janssen et al. (28)
55-64 yo	0.853	
65-74 yo	0.810	
75+ yo	0.735	
Uncontrolled		
45-54 yo	0.733	Rowles et al. (29)
55-64 yo	0.675	
65-74 yo	0.644	
75+ yo	0.584	
Disutility values		
Diabetes mellitus	0.190	Sullivan et al. (30)
Gastro-intestinal disorders	0.054	
Pegvisomant daily injection	0.023	Boye et al. (31)

Yo, Year Old.

Disutilities associated with adverse events were considered since diabetes mellitus and gastro-intestinal disorders could have an impact on patients’ quality of life (30). In addition, for pegvisomant which was daily-injected, a utility decrement was also applied (31).

### Costs

The analysis was performed from the collective perspective, where all the resources consumed in the production of the study interventions were valued, whatever the source of funding (patients, compulsory and supplementary health insurance schemes, the central government, etc.). All costs were expressed in Euros 2019.

Drug costs, including dispensing fees, were valued by prices obtained from the National Health Insurance Database (Base des medicaments et informations tarifaires de l’Assurance Maladie [BdM_IT]). Costs per year were calculated using doses and frequencies of administration obtained from the literature (21, 24). For pegvisomant, the cost of 15 mg was applied during the first year of the analyses and then the cost of 20 mg was applied, to reflect the real life use, based on Chanson et al. (24). A nursing cost was added for pasireotide and FGSA which had to be administered by a healthcare professional. Only the first dose of pegvisomant generally required a professional intervention. Finally, monitoring imaging and biological tests frequencies were based on recommendations and French clinical experts’ opinion and unit costs were obtained from French referentials (**Supplementary Table 1**). Only resources which were different between treatments were included in the analyses, therefore, no general practitioner or endocrinologist consultations were taken into account, assuming that there are similar across treatment strategies.

According to clinical expert, grade 1-2 gastro-intestinal disorders and grade 1-2 diabetes occurred along the time horizon. Grade 3-4 adverse event were considered as acute events and they were valued as a hospitalization based on the Echelle Nationale des Coûts (ENC) and weighted by public and private French hospitals repartition from ScanSanté. For health states costs, the incidence rates of the most frequent comorbidities in acromegaly for controlled and uncontrolled patients were valued using the literature. Cost inputs are reported in **Table 5**.

**Table 5:** Cost inputs.

Item	French use (%)	Unit	Unit cost (€)	Total cost (€)	Source
Drug cost for 2^nd^ line		Dose	Cost per mg	Cost per year	
Pegvisomant	100%	15mg/20mg^┼^	6.20€/6.13€	33,853.09€/44,606.77€	Chanson et al. (24) - BdM_IT
Pasireotide	100%	50.1mg	57.90€	37,712.31€	Gadelha et al. (21) - BdM_IT
Pegvisomant and FGSA					
Pegvisomant	100%	15mg	6.20€	33,853.09€	Trainer et al. (20) - BdM_IT
Octreotide	30%	30mg	20.51€	7,999.64€	Assumption - BdM_IT
Lanreotide	70%	120mg	12.07€	18,831.78€	Assumption - BdM_IT
FGSA					
Octreotide	30%	30mg	20.51€	7,999.64€	Assumption - BdM_IT
Lanreotide	70%	120mg	12.07€	18,831.78€	Assumption - BdM_IT
**Administration cost for 2^nd^ line**		**Number of visits**	**Cost per act**	**Cost per year**	
Pegvisomant	100%	1	7.00€	7.00€	Clinical expert opinion - NGAP
Other treatments	100%	13	7.00€	91.00€	
**Monitoring cost for 2^nd^ line╪**				**Cost per year**	
Pegvisomant					Clinical expert opinion
1^st^ year				611.72€	
Subsequent years				285.88€	
Pasireotide					
1^st^ year				598.56€	
Subsequent years				343.73€	
Pegvisomant and FGSA					
1^st^ year				483.12€	
Subsequent years				390.70€	
FGSA					
1^st^ year				483.12€	
Subsequent years				390.70€	
**Health state costs for 2^nd^**	**% in controlled**	**% in uncontrolled**	**Cost per year**	**Source**	
Cardiomyopathy	3.80%	7.30%	2,262.65€	Carmichael et al. (32) - Tuppin et al. (33)	
Hypertension	41.80%	58.50%	2,039.14€	Carmichael et al. (32) - Mennini et al. (34)	
Diabetes Mellitus	25.30%	41.50%	4,076.69€	Carmichael et al. (32) - Lagasnerie and al (35).	
Vertebral fracture	33.00%	80.00%	6,226.57€	Bonadonna et al (36) - Cotté et al. (37)	
Arthropathy	50.00%	70.00%	4,667.74€	Zhang et al. (38) - Guillemin and al (39).	
Obstructive sleep apnea	39.00%	56.00%	1,353.28€	Davi et al. (40) - Poulle and al (41).	
**Management of adverse events cost**		**Grade 1-2**	**Grade 3-4**	**Source**	
Diarrhea		45.81€	–	Mickish et al. (42)	
Nausea		81.56€	–		
Flatulence		45.81€	–		
Abdominal Pain		–	973.41€	ENC 2017	
Hyperglycemia		–	1,548.42€	ENC 2017	
Diabetes mellitus		4,076.69€	6,158.37€	Lagasnerie and al (35). - ENC 2017	

^╪^ A dose of 15mg per day was assumed during the first year of the time horizon and this dose was increased to 20mg per day for the subsequent years.

BdM_IT, Base des médicaments et informations tarifaires de l’Assurance Maladie; ENC, Echelle Nationale des Coûts; FGSA, First-generation somatostatin analogs; NGAP, Nomenclature générale des actes professionnels.

For health states costs, the incidence rates of the most frequent comorbidities in acromegaly for controlled and uncontrolled patients were valued using the literature (32–41). Cost inputs are reported in **Table 5**.

### Incremental Cost-Utility Ratio and Cost-Utility Frontier

To compare strategies in terms of cost-utility, the model aggregated the total costs and patient outcomes and the incremental cost utility ratio (ICUR) was estimated as follows:


$$ICUR=\frac{C_{a}-C_{b}}{QALY_{a}-QALY_{b}}$$

where C_a_ and C_b_ are respectively the total costs of the A and B strategy and QALY_a_ and QALY_b_ are respectively the total QALY of the A and B strategy.

The cost-utility frontier represents all situations for which there are no other interventions that provide a better health outcome at a lower cost (non-dominated interventions). Two types of dominances are considered:

- strict dominance: situation in which a treatment strategy is less costly than its comparator for identical or higher effectiveness level, or situation in which a strategy is more effective than its comparator for an identical or lower cost;
- weak dominance: a treatment is excluded by weak dominance if its ICUR relative to the next less costly, undominated alternative is greater than that of a more costly alternative.

### Sensitivity Analysis

Deterministic sensitivity analysis (DSA) was conducted to identify the key model drivers. Input values were varied one at a time to show their impact on model results (**Supplementary Table 2**). A probabilistic sensitivity analysis (PSA) was conducted to assess the robustness of model results. Random values were generated based on statistical distributions as follows:

- for SMRs, lognormal distributions were used;
- for utilities, proportion of patients with comorbidities, beta distributions were used;
- for costs and doses, gamma distributions were used;
- draws from CODA (Convergence Diagnostics and Output Analysis) of the NMA were used for the hazard ratios.

Then, 1,000 simulations of the model were run and for each simulation, net monetary benefits (NMB) were calculated for different values of the willingness-to-pay for one QALY for all treatments.


$$NMB=\lambda (QALY_{a}-QALY_{b})\times (C_{a}-C_{b})$$

where *λ* is the willingness-to-pay for one QALY.

Finally, the probability of being the most-effective (treatment with the highest NMB) was estimated on the 1,000 estimations.

Finally the following scenarios have been conducted:

- Scenario 1: no treatment strategy was added in the analysis;
- Scenario 2: 15 mg/day dose for pegvisomant along the time horizon;
- Scenario 3: societal perspective, including productivity losses;
- Scenario 4: alternative impact of IGF-1 controlled on comorbidities (43);
- Scenario 5: no additional cost and disutility for diabetes;
- Scenario 6/7: 10 year-time horizon and 20-year time horizon;

## Results

### Base Case

Treatment with FGSA generated the lowest cost and the lowest number of QALYs (467,433 € and 12.71) over 40 years (**Table 6**). On the contrary, treatment with pegvisomant in combination with FGSA generated the highest costs (1,229,168 €) and the highest number of QALY (16.80). For all treatments, the cost is mostly due to the drug acquisition.

**Table 6:** Costs, LY and QALY.

	FGSA	Pasireotide	Pegvisomant	Pegvisomant + FGSA
Drug acquisition costs	309,476€	783,787€	978,883€	1,009,046€
Drug administration costs	1,809 €	1,891€	7€	2,024€
Monitoring costs	7,890€	7,424€	6,682€	8,812€
Adverse events costs	3,699€	17,950€	251€	0 €
Health states costs	144,560€	134,931€	120,148€	119,286 €
**Total costs**	**467,433€**	**945,984€**	**1,105,971€**	**1,229,168€**
**Life Years**	**18.98**	**19.89**	**21.26**	**21.34**
**Quality Adjusted Life Years**	**12.71**	**13.56**	**16.44**	**16.80**

FGSA, First-generation somatostatin analogs.

Bold values are total results.

Incremental cost-effectiveness and cost-utility ratios were estimated for pasireotide, pegvisomant and pegvisomant + FGSA versus FGSA alone. Pasireotide treatment versus FGSA had an incremental benefit of 0.90 LY and 0.85 QALY with an incremental cost of 478,551 €. Therefore, its ICER was 529,496 € per LY gained and its ICUR was 562,463 € per QALY gained. For pegvisomant, the incremental benefit was 2.28 LY and 3.73 QALY with an incremental cost of 638,538 €. Therefore, the ICER and the ICUR of pegvisomant versus FGSA were lower than the one of pasireotide (**Table 7**).

**Table 7:** ICER and ICUR of the base case.

	ICER (cost per LY gained)	ICUR (cost per QALY gained)
Pasireotide versus FGSA	529,496€	562,463€
Pegvisomant versus FGSA	280,275€	171,332€
Pegvisomant + FGSA versus FGSA	323,035€	182,242€

ICER, Incremental cost-effectiveness ratios; ICUR, Incremental cost-utility ratios.

**Figure 2:**
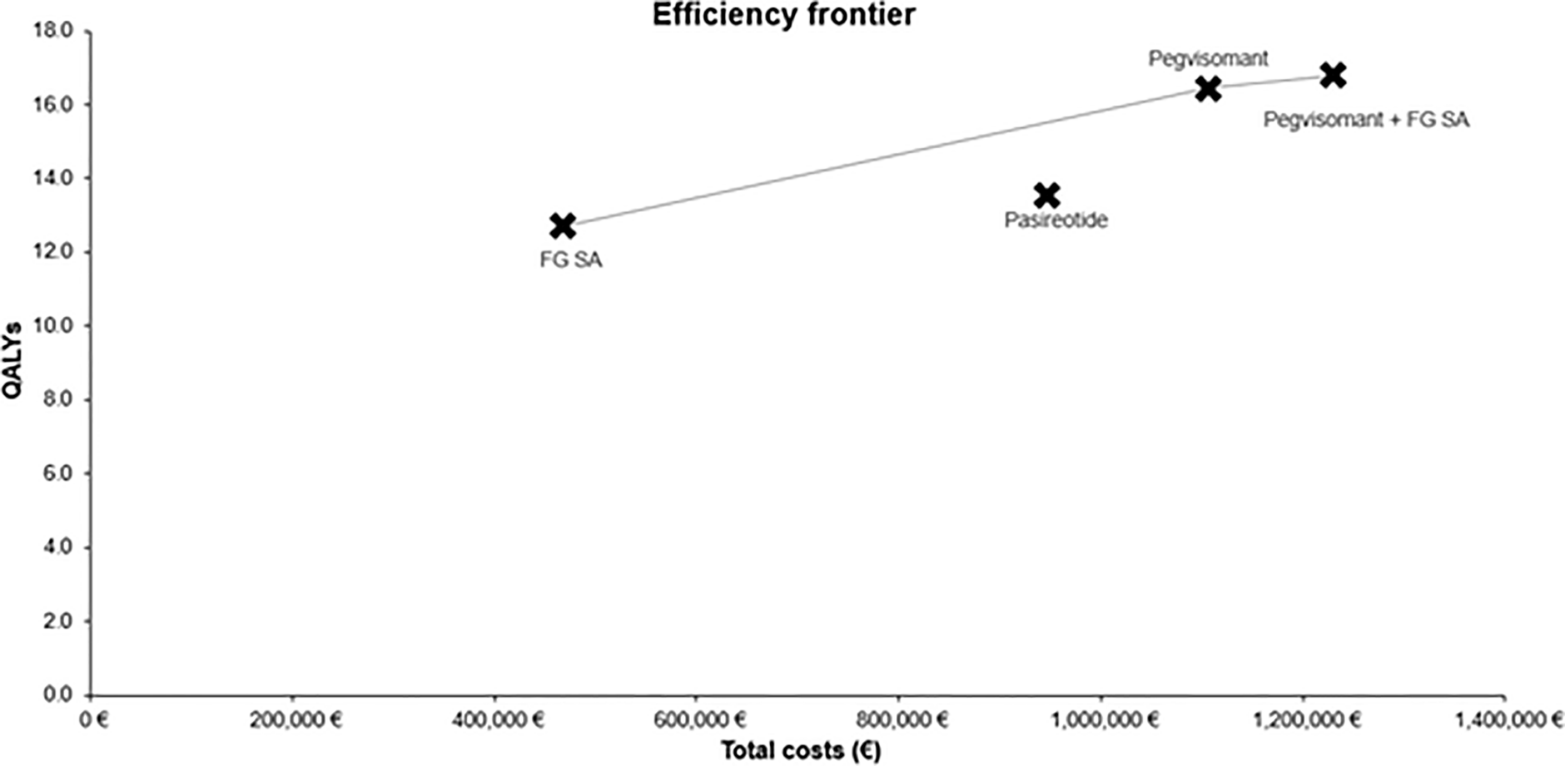
Cost-utility frontier of the base case analysis.

The cost-utility frontier was formed by FGSA, pegvisomant in monotherapy and pegvisomant in association with FGSA (**Figure 2**).

### Sensibility Analysis

The DSA was run for pegvisomant versus FGSA and pegvisomant and FGSA versus pegvisomant (**Supplementary Figures 1** and **2**). All DSA showed that the discount rates for costs and QALY and SMR for uncontrolled patients had the largest impact on the ICUR of treatments. The impact of discount rates was explained by the fact that patients on pegvisomant lived longer, therefore, the future costs and QALY had a higher impact on results.

**Figure 3:**
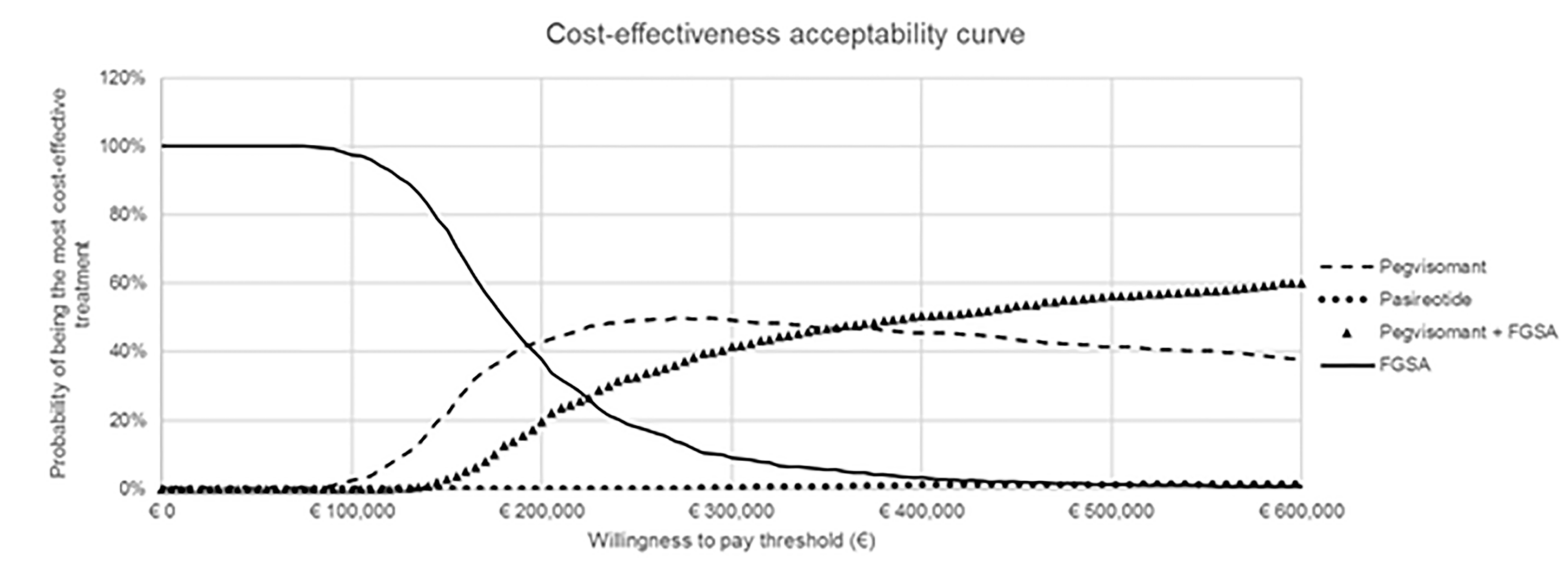
Cost-effectiveness acceptability curve.

In the PSA, the acceptability curve showed the probability of one intervention to be judged as “cost-effective” for different willingness-to-pay for an additional QALY values (**Figure 3**). FGSA was the most cost-effective strategy for a willingness-to-pay lower than 195,000 €. From a threshold of 195,000€ to 360,000 €, pegvisomant was likely the most cost-effective treatment option. Finally, above 360,000 €, pegvisomant in combination with FGSA was the optimal option. Across the 1,000 simulations of the PSA, pasireotide was always dominated by other strategies, therefore, its probability of being cost-effective is 0% regardless the willing-to-pay for an additional QALY.

### Scenario Analyses

Several scenario analyses have been conducted. In the first scenario, where a no treatment strategy was included, FGSA and pasireotide were not on the frontier, since they were weakly dominated by pegvisomant. In the scenario where a dose of 15 mg per day for pegvisomant was assumed along the time horizon, the order of treatments by increasing total cost has varied. Indeed, for a time horizon of 40 years, pegvisomant was less expensive than pasireotide. In this scenario, all treatments are evaluated on the same basis, with constant drug acquisition costs overtime, based on the dose received during the first year. Therefore, pasireotide was strictly dominated by pegvisomant. Results of this scenario are reported in **Table 8**.

**Table 8:** Scenario 2 analysis.

	Costs (€)	QALYs	Δ Costs (€)	Δ Effectiveness (QALYs)	ICUR (€/QALYs)
FGSA	467,433€	12.71			
Pegvisomant	877,615€	16.44	410,182€	3.73	110,060€
Pasireotide	945,984€	13.56	68,369€	-2.88	SD
Pegvisomant and FGSA	1,229,168€	16.80	283,184€	3.24	968,127€

ICUR, Incremental cost-utility ratios; QALY, Quality-adjusted life year; SD, Stricly dominated.

Other scenario analyses have slightly impacted the results (**Supplementary Table 3**).

## Discussion

Second-line pharmacological treatments for acromegaly have been recommended for several years now. Efficacy of these treatments has been assessed in many randomized clinical trials and real-world studies. Based on all these studies, it was shown that FGSA has a very low efficacy for these patients and pegvisomant with FGSA (off-label) was the most effective, in terms of IGF-1 normalization rates. No French study has evaluated if these treatment efficacy differences have an economic impact. Therefore, this study aimed to assess the cost-utility of these treatments.

The base case analysis, showed that FGSA, pegvisomant in monotherapy and pegvisomant in combination with FGSA were on the cost-utility frontier and pasireotide was weakly dominated by pegvisomant in monotherapy. These results were mainly due to the better control of patients with pegvisomant, in monotherapy or in association with FGSA, compared to pasireotide. Even if the drug acquisition cost of pegvisomant is higher (due to the higher unit cost and the longer treatment duration), better control of patients is associated with higher survival and quality of life, generating more QALY. In addition, fewer comorbidities occurred, therefore, health state costs are lower. Finally, a high percentage of patients developed diabetes due to pasireotide treatment, which is associated with loss of quality of life and healthcare costs.

This study was the first cost-utility analysis from a French collective perspective. All methodological assumptions in the model were based on Haute Autorité de Santé recommendations for health economic evaluation. In addition, our analysis was the first which included all clinically recommended treatments and comparison of efficacy based on a network-meta-analysis. This allowed to provide adjusted direct and indirect comparisons between treatments in the model.

Our results are consistent with the results of a cost-utility analysis on pegvisomant performed from a Polish perspective (12). The ICUR of pegvisomant compared to FGSA was estimated at 165,986 € per QALY gained in this study. However, this model only included pegvisomant and FGSA. Another health economic evaluation was published recently (14). This Spanish model reported an ICUR of 551,405 € per QALY gained for pasireotide and 85,869 € per QALY gained for pegvisomant compared to FGSA. The results of this model differed from our analysis since only pegvisomant efficacy was based on IGF-1 control and pasireotide and FGSA efficacies were based on IGF-1 and GH control.

The main limitation of the model was its binary structure. Indeed, two health states were modelled: controlled or uncontrolled patients. In real-world practice, the choice of treatment is not based on the IGF-1 control, but on the IGF-1 level. Therefore, the treatment and the dosing of each drug will vary more over time.

Then, acromegaly follow-up costs were included in the model, specific to health states. These costs were estimated based on the healthcare of comorbidities of acromegaly and the percentage of patients with comorbidities among controlled and uncontrolled patients. However, patients with acromegaly have often several comorbidities and health resources can be combined for the follow-up of these comorbidities. Therefore, these costs in the model were probably overestimated.

Since in France, no threshold for cost-effectiveness has been explicitly defined for efficiency, this health economic evaluation is only a complementary informative tool, in addition to the recommendations and treatment guidelines to help physicians in their prescribing decision, depending on patient and disease profile (especially regarding tumor concern and impaired glucose metabolism) (11).

## Data Availability Statement

The original contributions presented in the study are included in the article/**Supplementary Material**. Further inquiries can be directed to the corresponding author.

## Author Contributions

All authors contributed to the article and approved the submitted version. LM and LJ contributed to the development of the analysis.

## Funding

This study is sponsored by Pfizer Inc. Analysis and Medical writing support was provided by Steve Consultant and funded by Pfizer Inc. The authors declare that this study received funding from Pfizer. The funder was not involved in the study design, collection, analysis, and interpretation of data, the writing of this article or the decision to submit it for publication.

## Conflict of Interest

TB received research grants from Ipsen, Pfizer, and speaker fees from Advanz, Ipsen, Novartis, Pfizer, Strongbridge. He was a clinical trial investigator for Novartis, Strongbridge. PC has received unrestricted research and educational grants from Ipsen, Novartis, Novo-Nordisk, and Pfizer. He has served as an investigator (principal or coordinator) for clinical trials funded by Novartis, Pfizer, Ipsen, Italpharmaco, Antisense, and Prolor Biotech. He is an advisory board member for Ipsen, Novartis, Pfizer, and Teburio. He gave lectures for Ipsen, Novartis, and Pfizer. All the fees and honoraria are paid to his institution. PR has served on advisory boards Pfizer, Novartis, as investigator for clinical trials sponsored by Pfizer, Novartis, Sandoz, Novo Nordisk, has given lectures for Pfizer, IPSEN, Sandoz, has received unrestricted research or educational grants from Pfizer, Novartis, Novo Nordisk BD has served on advisory boards Pfizer, Novartis, and was speaker for Novartis, Ipsen, Pfizer. DD has served as an investigator for clinical trials funded by Novartis, has served on advisory boards Pfizer and gave lectures for Ipsen, Novartis, and Pfizer. LM and LJ are Steve Consultant employees RH and LS are Pfizer employees. GR has received unrestricted research grants from Ipsen and Novartis, GR has served as an investigator (principal or coordinator) for clinical trials funded by Novartis, Pfizer, Ipsen, Chiasm. He gave lectures for Ipsen, Novartis, and Pfizer.

## Publisher’s Note

All claims expressed in this article are solely those of the authors and do not necessarily represent those of their affiliated organizations, or those of the publisher, the editors and the reviewers. Any product that may be evaluated in this article, or claim that may be made by its manufacturer, is not guaranteed or endorsed by the publisher.
