# Supplementary material for: Cost-Utility of Acromegaly Pharmacological Treatments in a French Context
Source: Front Endocrinol (Lausanne). 2021 Oct 7;12:745843. doi: 10.3389/fendo.2021.745843 (PMC8531881; doi:10.3389/fendo.2021.745843)
Supplement: Supplementary file 1 [file DataSheet_1.pdf]

Supplementary Material

Supplementary Table 1. Frequencies and costs of monitoring imaging and biological tests

**Treatments**	**Monitoring exam**	**Frequency during the first year**	**Frequency during subsequent year**
Pegvisomant	ALAT and ASAT	6	X
	MRI	2	1
	IGF-1 level	4	2
Pasireotide	ALAT and ASAT	7	2
	FPG	15	X
	Glycated hemoglobin	15	X
	Abdomen ultrasound	1	1
	Electrocardiography	1	X
	MRI	1	1
	GF-1 level	4	2
Octreotide / Lanreotide	ALAT and ASAT	6	X
	Abdomen ultrasound	2	2
	MRI	1	1
	IGF-1 level	4	2
Pegvisomant + FGSA	ALAT and ASAT	6	X
	Abdomen ultrasound	2	2
	MRI	1	1
	IGF-1 level	4	2
**Type of resources**			**Unit cost (€)**
ALAT and ASAT			2.70†
FPG			1.35†
Glycated hemoglobin			5.94†
Abdomen ultrasound			52.45
Electrocardiography			14.26
MRI			233.50
IGF-1 level			20.25†

ALAT: Alanine aminotransferase; ASAT; Asparate aminotransferase ; FPG : Fasting plasma glucose ; IGF-1: Insulin-like growth factor-1 ; MRI: Magnetic resonance imaging ;

† *For laboratory tests, a security package for the treatment of a blood sample and a pre-analytical patient care package were added to unit costs.*

Supplementary Table 2. Determinist sensibility analysis inputs

**DSA parameters**	**Base Case**	**Min**	**Max**	**Source**
**Patients characteristics**				
Age at treatment initiation – 2nd line	47.0	37.6	56.4	+/- 20%
% of males – 2nd line	50.7%	40.5%	60.8%	+/- 20%
Age at treatment initiation – 3rd line	53.0	42.4	63.6	+/- 20%
% of males – 3rd line	52.5%	42.0%	63.0%	+/- 20%
**Mortality**				
SMR controlled patients	1.1	0.9	1.4	Holdaway et al. 2008
SMR uncontrolled patients	2.5	1.6	4.0	
**Health state costs**				
Cardiomyopathy - % controlled	3.8%	3.0%	4.6%	+/- 20%
Cardiomyopathy - % uncontrolled	7.3%	5.8%	8.8%	+/- 20%
Cardiomyopathy – annual cost	2,262.65€	1810,12€	2715,18€	+/- 20%
Hypertension - % controlled	41.8%	33.4%	50.2%	+/- 20%
Hypertension - % uncontrolled	58.5%	46.6%	70.2%	+/- 20%
Hypertension – annual cost	2,039.14€	1,631.31€	2,446.97€	+/- 20%
Vertebral fracture - % controlled	33.0%	26.4%	39.6%	+/- 20%
Vertebral fracture - % uncontrolled	80.0%	64.0%	96.0%	+/- 20%
Vertebral fracture – annual cost	6,226.57€	4,981.26€	7,471.88€	+/- 20%
Arthropathy - % controlled	50.0%	40.0%	60.0%	+/- 20%
Arthropathy - % uncontrolled	70.0%	56.0%	84.0%	+/- 20%

DSA parameters	Base Case	Min	Max	Source
Arthropathy – annual cost	4,667.74€	3,734.19€	5,601.29€	+/- 20%
Diabetes mellitus - % controlled	25.3%	20.2%	30.4%	+/- 20%
Diabetes mellitus - % uncontrolled	41.5%	33.2%	49.8%	+/- 20%
Diabetes mellitus – annual cost	4,076.69€	3,261.35€	4,892.03€	+/- 20%
Obstructive sleep apnea - % controlled	39.0%	31.2%	46.8%	+/- 20%
Obstructive sleep apnea - % uncontrolled	56.0%	44.8%	67.2%	+/- 20%
Obstructive sleep apnea – annual cost	1,353.28€	1,082.62€	1,623.94€	+/- 20%
**Quality of life**				
44-54 yo - controlled	0.922	0.811	0.986	+/-5%
44-54 yo - uncontrolled	0.733	0.658	0.802	+/-5%
55-64 yo - controlled	0.853	0.760	0.926	+/-5%
55-64 yo - uncontrolled	0.678	0.610	0.743	+/-5%
65-74 yo- controlled	0.810	0.725	0.883	+/-5%
65-74 yo - uncontrolled	0.644	0.580	0.706	+/-5%
75+ yo- controlled	0.735	0.660	0.804	+/-5%
75+ yo - uncontrolled	0.584	0.526	0.641	+/-5%
**Model settings**				
Discounting - cost	2.5%	0.0%	4.0%	HAS
Discounting - QALY	2.5%	0.0%	4.0%	
**Treatment efficacy**				
Efficacy – Pasireotide 3 months	12.7%	6.9%	19.8%	95% CI of the beta distribution
Efficacy – Pasireotide 6 months	25.4%	18.3%	30.9%	LB: 95% CI of the beta distribution<br>UB: efficacy at 9 months
Efficacy – Pasireotide 9 months	30.9%	25.4%	33.0%	LB: efficacy at 6 months<br>UB: efficacy at 12 months
Efficacy – Pasireotide 12 months	33.0%	30.9%	43.1%	LB: efficacy at 9 months<br>UB: 95% CI of the beta distribution
Efficacy - pegvisomant + pasireotide 3 months	33.7%	24.8%	43.2%	95% CI of the beta distribution
Efficacy - pegvisomant + pasireotide 6 months	67.4%	53.4%	80.0%	LB: 95% CI of the beta distribution<br>UB: efficacy at 9 months
Efficacy - pegvisomant + pasireotide 9 months	69.6%	60.2%	78.1%	LB: 95% CI of the beta distribution<br>UB: efficacy at 12 months
Efficacy - pegvisomant + pasireotide 12 months	71.7%	58.1%	83.6%	LB: efficacy at 9 months<br>UB: 95% CI of the beta distribution

Supplementary Table 3. Scenario results

	Scenario 3	Scenario 4	Scenario 5	Scenario 6	Scenario 7
**FGSA**	-	-	-	-	-
**Pasireotide**	WD	WD	WD	WD	WD
**Pegvisomant**	151,798€	168,698€	180,254€	228,447€	206,715€
**Pegvisomant and FGSA**	332,171€	338,315€	339,267€	423,441€	374,404€

FGSA: First generation somatostatin analogs; WD: Weakly dominated.

Supplementary Figure 1. Tornado plot of the DSA for pegvisomant versus FGSA

Supplementary Figure 2. Tornado plot of the DSA for pegvisomant + FGSA versus pegvisomant
