# Supplementary figures and images for: Cost-Utility of Acromegaly Pharmacological Treatments in a French Context
Source: Front Endocrinol (Lausanne). 2021 Oct 7;12:745843. doi: 10.3389/fendo.2021.745843 (PMC8531881; doi:10.3389/fendo.2021.745843)

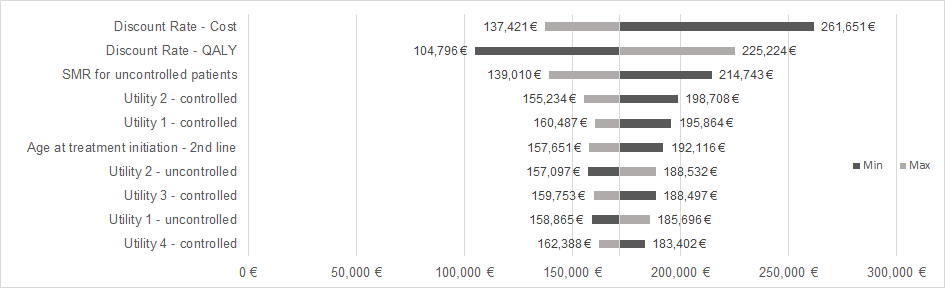

Supplement: Supplementary file 2 [file Image_1.tif]

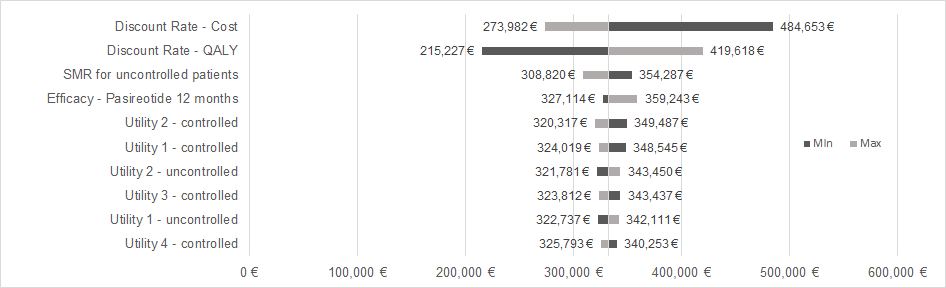

Supplement: Supplementary file 3 [file Image_2.tif]
